# A study on risk prediction of decline in self-care ability one month after discharge in postoperative colorectal cancer patients based on routine clinical indicators

**DOI:** 10.3389/fsurg.2026.1863229

**Published:** 2026-06-05

**Authors:** Wenjie Wang, Fu Yang, Xian Shen, Yifan Jiang, Rui Tai, Yangyang Li, Yan Peng, Pengfei Yang, Yanping Zhuang, Jiaqi Yu, Mei Wang, Fang Fang

**Affiliations:** 1 Shanghai General Hospital, Shanghai Jiao Tong University School of Medicine, Shanghai, China; 2 Department of Nursing, Yangpu District Shidong Hospital of Shanghai, Yangpu, China; 3 School of Nursing, Shanghai Jiao Tong University, Shanghai, China

**Keywords:** activities of daily living, colorectal neoplasms, NURSING, postoperative rehabilitation, predictive model

## Abstract

**Objective:**

To investigate the risk factors for the decline in activities of daily living (ADL) one month after discharge in colorectal cancer patients with normal ADL at discharge, and to establish a risk prediction model, so as to provide evidence for early screening of high-risk populations and optimized transitional care in clinical practice.

**Methods:**

A single-center retrospective cohort study was conducted. A total of 320 colorectal cancer patients who underwent surgery at the COC Diagnosis and Treatment Center of Shanghai General Hospital from January 2023 to December 2025, with grade A Barthel Index at discharge and completed one-month follow-up, were enrolled. Demographic data, underlying diseases, tumor and treatment information, and laboratory indicators were collected. Univariate and multivariate Logistic regression analyses were used to screen independent risk factors. A prediction model was constructed and internally validated using the Bootstrap method.

**Results:**

Among the 320 patients, 62 (19.4%) developed ADL decline one month after discharge. Multivariate analysis revealed that height and retinol-binding protein were independently negatively correlated with reduced self-care ability (protective associations), while mean red blood cell volume, triglycerides, and coronary heart disease history were independently positively correlated (risk associations). All differences were statistically significant (*P* < 0.05). The prediction model based on the above five indicators had an apparent AUC of 0.884 and a corrected AUC of 0.875, showing good discrimination and calibration.

**Conclusion:**

The prediction model incorporating height, mean corpuscular volume, retinol-binding protein, triglyceride, and coronary heart disease history can effectively identify high-risk patients early and provide a quantitative reference for discharge preparation and transitional care.

## 1 Introduction

Colorectal cancer is one of the most common malignant tumors of the digestive system. Surgical treatment remains the core modality for most patients to achieve radical cure or staged disease control (1). With advances in perioperative management, anesthesia techniques and minimally invasive surgery, perioperative mortality and the incidence of severe complications in patients with colorectal cancer have decreased significantly compared with previous eras. Clinical focus has gradually shifted from mere survival outcomes to postoperative functional recovery and improvement in quality of life (2). For patients with colorectal cancer, surgical trauma, nutritional depletion, tumor burden, comorbid conditions and perioperative stress responses often jointly affect their postoperative recovery process. Even after uneventful hospital discharge, patients may still experience short-term declines in exercise tolerance, physical capacity and impaired activities of daily living (ADL) (3). Therefore, hospital discharge does not equate to the completion of functional recovery. The early postoperative period, especially within one month after discharge, remains a critical stage characterized by fluctuations in patients' functional status and changes in care needs (4).

ADL serve as an important indicator reflecting patients' basic functional status, independent living ability, and rehabilitation level (5). Compared with traditional outcomes such as complications, readmission, or length of hospital stay alone, ADL can more directly reflect patients' actual ability to return to daily life after surgery, and has direct clinical significance for evaluating rehabilitation quality, care burden, and the need for transitional care. As a commonly used clinical ADL assessment tool, the Barthel Index shows favorable operability and application foundation, and has been widely adopted for functional evaluation in postoperative patients and populations with chronic diseases (6). For patients after colorectal cancer surgery, a short-term decline in ADL after discharge may not only indicate insufficient recovery and persistent frailty, but also be associated with increased subsequent care needs, heavier family care burden, delayed rehabilitation progress, and elevated risk of adverse prognosis (7). Therefore, conducting research on changes in self-care ability during the early post-discharge period has clear clinical value.

Most existing studies on the prognosis of patients after colorectal cancer surgery have focused on postoperative complications, oncological outcomes, nutritional status, quality of life, or long-term survival, whereas research on the risk of short-term decline in ADL after discharge remains relatively limited (8, 9). In clinical practice especially, some patients appear to be in stable general condition at discharge and may even possess basic self-care ability, yet after returning to the home environment, they may still experience a short-term decline in self-care ability due to underlying diseases, insufficient nutritional reserves, cardiovascular burden, metabolic abnormalities, inadequate postoperative physical recovery, insufficient care support, and other factors (10). Identifying high-risk patients before discharge using routine clinical information, and providing individualized follow-up, rehabilitation guidance, and transitional care interventions, may help improve their early postoperative functional recovery trajectory (4). Nevertheless, risk stratification tools for this population are still lacking, and simple and feasible prediction models for the early identification of high-risk patients remain scarce in clinical practice.

Accordingly, this study intends to enroll patients with normal ADL at discharge after colorectal cancer surgery as the study subjects. We aim to analyze the factors associated with the decline in self-care ability one month after discharge and establish a risk prediction model, so as to provide evidence for the early clinical screening of high-risk patients, optimization of discharge preparation, and management of transitional care.

## 2 Methods

### 2.1 Study design

This was a single-center retrospective cohort study aimed at identifying risk factors associated with the decline in ADL one month after discharge among patients with normal self-care ability at discharge following colorectal cancer surgery, and constructing a corresponding risk prediction model.

The study design and results reporting complied with the Strengthening the Reporting of Observational Studies in Epidemiology (STROBE) (11) statement. Reporting of the prediction model referred to the Transparent Reporting of a multivariable prediction model for Individual Prognosis Or Diagnosis (TRIPOD) (12) statement.

### 2.2 Study setting and participants

This study was based on the medical record management system and follow-up database of the COC Diagnosis and Treatment Center, Shanghai General Hospital. Clinical data were retrospectively collected from patients who underwent surgical treatment for colorectal cancer at this center between January 2023 and December 2025. The inclusion criteria were as follows: (1) age ≥18 years; (2) pathologically confirmed colorectal cancer; (3) underwent surgery for colorectal cancer and survived to discharge; (4) grade A on the Barthel Index at discharge; (5) completed follow-up assessment of the Barthel Index at 1 month after discharge. Exclusion criteria were as follows: (1) missing data on the main predictor variables; (2) missing outcome data at 1 month after discharge; (3) death within 1 month after discharge; (4) inability to complete the Barthel Index assessment at the 1-month follow-up after discharge due to disturbance of consciousness, critical illness, or other reasons, and being classified as Grade G.

### 2.3 Sample size calculation

As a single-center retrospective study for prediction model development, the sample size was determined by the number of available cases meeting the inclusion and exclusion criteria during the study period, and no dedicated *a priori* sample size calculation was performed before study initiation. A total of 320 patients were finally included, among whom 62 had the outcome event. The final model comprised 5 predictive parameters, yielding an events-per-variable (EPV) ratio of approximately 12.4, which exceeded the conventional empirical criterion of EPV ≥10. However, considering that a considerable number of candidate variables were examined during model development and that the EPV method is only a rough empirical rule, the risk of overfitting may still exist. Therefore, the bootstrap method was used for internal validation in this study, and the results still require further validation in independent samples.

### 2.4 Main outcome variable

The primary outcome of this study was whether patients experienced a decline in ADL one month after discharge. The ADL classification was used as the main outcome indicator. In this study, the Barthel Index was adopted to evaluate the rating scale, which is currently an internationally recognized gold standard for ADL assessment. Its reliability and validity have been fully verified in the population, and it is suitable for evaluating the self-care ability of patients with chronic diseases and postoperative patients.

1. Scale content and scoring criteria

The Barthel Index consists of 10 items of ADL, including feeding, grooming, dressing, toileting, transfer between bed and chair, walking on level ground, climbing stairs, bowel control, bladder control, and bathing. Each item is scored 0, 5, or 10 points according to the patient's level of independence (some items are further subdivided based on the degree of assistance required). The total score ranges from 0 to 100, with a higher score indicating better ability to perform ADL independently.

(2) Classification criteria for self-care

Ability According to the total Barthel Index score, patients' self-care ability was classified into seven grades as follows:

Grade A (complete independence): total score ≥95, able to perform all daily activities independently without assistance;
Grade B (mild dependence): total score 75–94, requiring minimal assistance for only a few complex activities;
Grade C (moderate dependence): total score 50–74, requiring assistance for multiple daily activities;
Grade D (severe dependence): total score 25–49, requiring full assistance for most daily activities;
Grade E (extremely severe dependence): total score 10–24, almost unable to perform any daily activities independently;
Grade F (total dependence): total score <10, completely unable to perform self-care and requiring 24-h professional care;
Grade G (unassessable): patients unable to complete the scale assessment due to impaired consciousness, critical illness, or other reasons.

According to the outcome definition of this study, a total Barthel Index score ≥95 at one month after discharge was defined as normal ADL (Grade A), and a total score <95 was defined as impaired ADL (Grades B–F). Since valid ADL outcome assessment could not be obtained for patients with Grade G, they were excluded from the outcome analysis.

In this study, Grade A was assigned a value of 0 and Grades B–F a value of 1 to construct a binary dependent variable for subsequent risk factor analysis and predictive model development.

### 2.5 Predictive variables

Based on previous literature and clinical availability, candidate predictive variables were initially included in this study, covering demographic and physical characteristics, lifestyle and comorbidity history, tumor and treatment-related information, as well as laboratory test indicators. All candidate predictive variables were derived from clinical data available before discharge, ensuring their temporality preceded the outcome assessment at 1 month after discharge.

1. Demographic and physical characteristics

Included sex, age, height, and weight. Relevant data were obtained from the initial nursing assessment records at admission.

(2) Lifestyle and comorbidity history

Included smoking history, drinking history, history of hypertension, diabetes mellitus, coronary heart disease, and cerebrovascular disease. Relevant data were obtained from medical records and admission assessment records.

(3) Tumor and treatment-related information

Included tumor stage, presence of other malignant tumors, length of hospital stay, and whether multiple surgeries were performed. Relevant data were obtained from operative records, postoperative medical records, pathological reports, and the front sheet of medical records.

(4) Laboratory test indicators

Fasting venous blood samples were collected by nursing staff at 6:00 on the day of discharge, and tests were performed by professional technicians in the Department of Laboratory Medicine. Data were obtained from the hospital laboratory information system. Indicators included:

1. Routine blood test

Hemoglobin, red blood cell count, hematocrit, mean corpuscular volume, mean corpuscular hemoglobin, mean corpuscular hemoglobin concentration, red blood cell distribution width, coefficient of variation of red blood cell distribution width, nucleated red blood cells, white blood cell count, neutrophils, lymphocytes, monocytes, eosinophils, basophils, platelet count, plateletcrit, mean platelet volume, coefficient of variation of platelet distribution width, platelet large cell ratio

2. Liver function

Total bilirubin, direct bilirubin, indirect bilirubin, total bile acid, total protein, albumin, globulin, albumin/globulin ratio, prealbumin, alanine aminotransferase, aspartate aminotransferase, mitochondrial aspartate aminotransferase isoenzyme, alkaline phosphatase, gamma-glutamyl transferase, lactate dehydrogenase, glutamate dehydrogenase

3. Renal function

Urea, creatinine, glomerular filtration rate, uric acid, retinol-binding protein

4. Blood glucose, electrolytes and blood lipids

Glucose, potassium, sodium, chloride, triglycerides

### 2.6 Data collection and quality control

This study was conducted after obtaining ethical approval. A pilot extraction was performed on 30 cases in the preliminary stage to standardize the data extraction process, variable definitions, and coding rules. Formal data were retrospectively extracted from the hospital electronic medical record system and the COC diagnosis and treatment platform using a standardized data extraction form by uniformly trained researchers.

Demographic data, comorbidity history, and perioperative information were obtained from medical records; physical data were derived from the initial nursing assessment on the day of admission; laboratory indicators were taken from the last fasting venous blood test results before discharge; and the primary outcome indicator was the Barthel Index assessment recorded at the 1-month follow-up after discharge.

To ensure data quality, two researchers independently cross-checked the extracted data. Logically inconsistent data, outliers, and missing information were verified by tracing the original medical records. Cases with missing key predictive variables or outcome variables were excluded, and complete-case analysis was adopted.

### 2.7 Bias control

To minimize selection bias, this study consecutively included all cases meeting the inclusion and exclusion criteria during the study period and applied uniform screening standards. To reduce information bias, all in-hospital predictive variables were derived from original records in the hospital electronic medical record system and the COC platform and extracted within a predefined time window; the outcome variable was uniformly defined as the Barthel Index assessment at 1 month after discharge.

To decrease errors in data extraction, data extractors received standardized training before the study, and double-checking was adopted for quality control. Given the large number of candidate variables and limited sample size, a multivariable Logistic regression model was constructed, and the bootstrap method was used for internal validation to evaluate model stability and potential overfitting risk.

### 2.8 Statistical analysis and model construction

Statistical analyses were performed using IBM SPSS Statistics 27.0. The outcome variable was whether a decline in self-care ability occurred one month after discharge: a total Barthel Index score ≥95 was defined as no decline in self-care ability and coded as 0; a total Barthel Index score <95 was defined as a decline in self-care ability and coded as 1.

Normality tests were first conducted for continuous variables. Those with a normal distribution were presented as mean ± standard deviation, and between-group comparisons were performed using independent-samples *t*-test. Non-normally distributed variables were expressed as median (interquartile range), and between-group comparisons were analyzed using the Mann–Whitney *U* test. Categorical variables were described as frequency and percentage, and between-group comparisons were carried out using the *χ*^2^ test or Fisher's exact test.

Based on univariate analysis, candidate variables were selected in combination with clinical significance for inclusion in the multivariable Logistic regression model. Continuous variables were entered into the model as raw values, and categorical variables were treated as dummy variables. Binary Logistic regression analysis was used to identify independent factors associated with a decline in self-care ability one month after discharge. Results were reported as regression coefficient (*β*), standard error (SE), Wald *χ*^2^ value, odds ratio (OR), and its 95% confidence interval (95% CI). Multicollinearity between variables was assessed during modeling.

All candidate variables with a univariate *P* value <0.05 were entered simultaneously into the multivariable logistic regression model using the Enter method (no automated stepwise selection was used). The final model retained only those variables that remained statistically significant after mutual adjustment. To assess the robustness of the model, a sensitivity analysis was conducted by forcing age and sex into the model together with the five original predictors, again using the Enter method. The stability of the odds ratios of the original predictors was examined.

The discriminative ability of the model was evaluated using the area under the receiver operating characteristic curve (AUC). Internal validation was performed using the Bootstrap resampling method to evaluate model stability (13). All statistical tests were two-sided, and *P* < 0.05 was considered statistically significant.

### 2.9 Ethical considerations

This study was approved by the Medical Ethics Committee of Shanghai General Hospital and conducted in accordance with the ethical principles of the Declaration of Helsinki.

Prior to the study, the purpose, procedures, confidentiality principles, and right to voluntary participation were fully explained to the patients, and written informed consent was obtained. Throughout the study, the principles of informed consent, voluntarism, information confidentiality, and non-maleficence were strictly adhered to protect patients' privacy and legitimate rights and interests.

## 3 Results

### 3.1 Analysis of baseline patient characteristics

**Figure 1:**
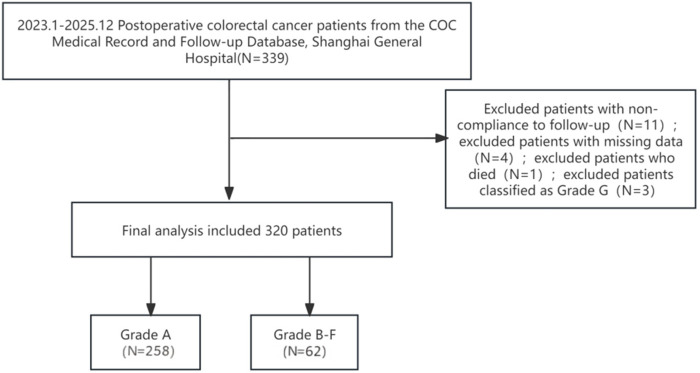
Patient selection flowchart.

A total of 339 postoperative colorectal cancer patients were initially enrolled from the medical record management system and follow-up database of the COC Diagnosis and Treatment Center, Shanghai General Hospital between January 2023 and December 2025. According to the exclusion criteria, 19 patients were excluded, including 4 with missing data, 1 who died, 11 who refused follow-up, and 3 who were graded as G. Finally, 320 eligible patients were included, among whom 258 were grade A and 62 were grades B–F (Figure 1). At discharge, all patients had a Barthel Index score of 97.53 ± 1.71 (mean ± SD), consistent with Grade A. At one-month follow-up, the 258 patients who remained independent (Grade A) had a mean Barthel score of 97.40 ± 1.59, while the 62 patients who experienced ADL decline (Grades B–F) had a mean Barthel score of 87.62 ± 19.81. Univariate analyses were performed on general data, laboratory indicators, physical indicators, underlying diseases, and treatment-related variables, stratified by whether a decline in ADL occurred at 1 month after discharge. The results showed statistically significant differences between the two groups in height, weight, mean corpuscular volume, mean corpuscular hemoglobin, coefficient of variation of platelet volume distribution width, indirect bilirubin, total bile acid, albumin/globulin ratio, aspartate aminotransferase, mitochondrial aspartate aminotransferase isoenzyme, alkaline phosphatase, gamma-glutamyl transferase, glutamate dehydrogenase, urea, retinol-binding protein, triglyceride, length of hospital stay, history of diabetes, history of coronary heart disease, presence of other malignant tumors, and multiple surgeries (all *P* < 0.05) (Table 1).

**Table 1:** Univariate analysis of risk factors affecting self-care ability in colorectal cancer patients.

Variable	Normal self-care ability (Grade A)	Decreased self-care ability (Levels B–F)	*t*/*χ*^2^/*Z* value	*P* value
Height (cm)	164.65 (158.00, 170.00)	160.95 (156.70, 165.13)	2.703	0.008
Weight (kg)	63.30 (57.00, 71.93)	60.05 (55.73, 63.38)	3.720	<0.001
Age	65.85 ± 11.93	67.71 ± 10.17	−1.130	0.259
Hemoglobin	116.58 ± 20.65	119.78 ± 21.71	−1.085	0.279
Red blood cell count	4.13 ± 0.58	3.98 ± 0.51	1.814	0.071
Platelet count	218.68 ± 79.39	213.15 ± 83.51	0.294	0.769
White blood cell count	6.88 ± 2.36	7.33 ± 2.06	−1.377	0.169
Neutrophils	71.98 ± 10.67	72.06 ± 9.72	−0.053	0.957
Lymphocytes	17.00 (11.17, 23.00)	17.54 (14.66, 19.94)	−0.520	0.604
Monocytes	6.79 ± 2.48	7.74 ± 2.68	−1.445	0.149
Eosinophils	2.60 ± 1.85	2.78 ± 1.56	−0.736	0.462
Basophils	0.31 ± 0.20	0.35 ± 0.18	−1.413	0.159
Hematocrit	34.98 ± 5.48	36.16 ± 4.66	−1.575	0.116
Mean corpuscular volume	87.07 (81.26, 91.95)	93.14 (88.66, 95.64)	−6.680	<0.001
Mean corpuscular hemoglobin	28.51 (26.00, 30.51)	29.42 (28.33, 30.58)	−2.654	0.009
Mean corpuscular hemoglobin concentration	332.00 (323.06, 340.22)	331.13 (322.93, 337.34)	0.943	0.348
Red cell distribution width	42.62 (38.84, 47.61)	44.64 (39.27, 52.99)	−1.691	0.095
RDW coefficient of variation	14.40 ± 5.20	15.11 ± 4.04	−1.002	0.317
Platelet distribution width coefficient of variation	16.35 (15.70, 17.22)	16.17 (15.99, 16.39)	3.379	0.001
Platelet large cell ratio	27.44 ± 9.67	28.76 ± 8.37	−0.995	0.321
Mean platelet volume	10.08 ± 1.79	10.20 ± 1.36	−0.493	0.622
Plateletcrit	0.22 ± 0.07	0.23 ± 0.07	−1.283	0.200
Nucleated red blood cells	0.02 ± 0.08	0.02 ± 0.04	−0.283	0.777
Total bilirubin	11.23 ± 6.66	12.51 ± 7.00	−1.345	0.179
Direct bilirubin	4.85 (2.90, 7.63)	5.35 (3.63, 6.90)	0.714	0.476
Indirect bilirubin	6.77 ± 3.53	8.03 ± 4.17	−2.431	0.016
Total bile acid	4.44 (1.75, 8.01)	2.53 (1.37, 4.29)	6.191	<0.001
Total protein	61.30 ± 6.05	60.53 ± 6.82	0.867	0.387
Albumin	35.16 ± 4.32	35.81 ± 5.03	−1.028	0.305
Globulin	24.70 (21.90, 28.70)	25.60 (23.33, 28.78)	−1.334	0.185
Albumin/globulin ratio	1.80 (1.30, 3.60)	1.38 (1.28, 1.48)	8.198	<0.001
Prealbumin	176.70 (143.58, 204.35)	157.21 (113.63, 218.51)	0.071	0.943
Alanine aminotransferase	30.93 ± 21.62	28.55 ± 17.20	0.805	0.422
Aspartate aminotransferase	34.50 (20.00, 58.00)	27.21 (20.47, 37.50)	4.692	<0.001
Mitochondrial AST isoenzyme	9.00 (5.00, 15.00)	8.00 (5.33, 11.25)	3.436	0.001
Alkaline phosphatase	95.50 (63.75, 128.00)	77.50 (67.75, 96.25)	3.925	<0.001
Gamma-glutamyl transferase	59.00 (24.00, 106.25)	41.41 (21.00, 62.31)	5.429	<0.001
Lactate dehydrogenase	184.00 (151.75, 215.00)	185.31 (156.50, 204.22)	0.246	0.806
Glutamate dehydrogenase	6.00 (3.00, 13.00)	5.00 (2.75, 7.81)	4.619	<0.001
Urea	7.45 (4.68, 13.53)	5.60 (4.20, 7.64)	5.624	<0.001
Creatinine	59.95 (50.46, 70.42)	61.68 (47.34, 73.44)	−0.249	0.804
Glomerular filtration rate	95.50 (86.68, 103.60)	90.13 (82.63, 99.27)	1.903	0.061
Uric acid	232.90 ± 85.47	211.70 ± 86.76	1.747	0.082
Retinol-binding protein (RBP)	32.39 ± 9.39	27.16 ± 10.22	3.869	<0.001
Glucose (Glu)	5.75 ± 1.73	5.40 ± 1.44	1.457	0.146
Potassium (K⁺)	4.06 ± 0.65	4.12 ± 0.49	−0.641	0.522
Sodium (Na⁺)	138.69 ± 2.88	139.46 ± 2.95	−1.876	0.062
Chloride (Cl⁻)	105.80 (103.08, 107.90)	106.20 (103.68, 109.97)	−1.263	0.210
Triglyceride (TG)	1.19 (0.84, 1.54)	1.57 (0.83, 3.79)	−4.701	<0.001
Length of hospital stay (d)	13.00 (8.00, 17.00)	14.50 (11.00, 18.25)	−2.067	0.041
Gender [*n* (%)]			0.042	0.838
Male	170 (65.9)	40 (64.5)		
Female	88 (34.1)	22 (35.5)		
Smoking [*n* (%)]			0.000	0.985
Yes	21 (8.1)	5 (8.1)		
No	237 (91.9)	57 (91.9)		
Alcohol drinking [*n* (%)]			0.432	0.511
Yes	15 (5.8)	5 (8.1)		
No	243 (94.2)	57 (91.9)		
Hypertension [*n* (%)]			0.493	0.483
Yes	108 (41.9)	29 (46.8)		
No	150 (58.1)	33 (53.2)		
Diabetes mellitus [*n* (%)]			35.211	<0.001
Yes	32 (12.4)	28 (45.2)		
No	226 (87.6)	34 (54.8)		
Coronary heart disease [*n* (%)]			62.736	<0.001
Yes	9 (3.5)	23 (37.1)		
No	249 (96.4)	39 (62.9)		
Cerebral infarction [*n* (%)]			0.089	0.766
Yes	18 (7.0)	5 (8.1)		
No	240 (93.0)	57 (91.9)		
Disease stage [*n* (%)]			0.126	0.723
Benign	41 (15.9)	11 (17.7)		
Malignant	217 (84.1)	51 (82.3)		
Complicated with other malignancies [*n* (%)]			15.258	<0.001
Yes	21 (8.1)	16 (25.8)		
No	237 (91.9)	46 (74.2)		
Multiple surgeries [*n* (%)]			6.415	0.011
Yes	158 (61.2)	27 (43.5)		
No	100 (38.8)	35 (56.5)		

### 3.2 Multivariate logistic regression analysis

The risk grouping of self-care ability in patients with colorectal cancer was used as the dependent variable (Group 1 = Grade A, Group 2 = Grades B–F). Variables with statistical significance in univariate analysis (*P* < 0.05) were included as independent variables, namely height, weight, mean corpuscular volume, mean corpuscular hemoglobin, coefficient of variation of platelet volume distribution width, indirect bilirubin, total bile acid, albumin/globulin ratio, aspartate aminotransferase, mitochondrial aspartate aminotransferase isoenzyme, alkaline phosphatase, gamma-glutamyl transferase, urea, retinol-binding protein, triglycerides, length of hospital stay, presence of other malignant tumors, history of diabetes mellitus, and history of coronary heart disease. Continuous variables were entered into the model as raw values, and dummy variables were set for categorical variables before performing binary Logistic regression analysis.

The final results of Logistic regression analysis showed that height, mean corpuscular volume, retinol-binding protein, triglycerides, and coronary heart disease were independent influencing factors for the decline in self-care ability in patients with colorectal cancer (*P* < 0.05) (Table 2).

**Table 2:** Multivariate analysis of influencing factors for self-care ability risk in colorectal cancer patients.

Variable	*B*	S.E.	Wald	Sig.	Exp (*B*)	95% CI for EXP(*B*)	
						Lower	Upper
Height	−0.072	0.031	5.442	0.020	0.931	0.876	0.989
Weight	−0.033	0.026	1.563	0.211	0.968	0.919	1.019
Mean Corpuscular Volume	0.101	0.041	6.062	0.014	1.106	1.021	1.198
Mean Corpuscular Hemoglobin	0.037	0.097	0.144	0.704	1.038	0.857	1.256
Platelet Volume Distribution Width Coefficient of Variation	−0.515	0.264	3.805	0.051	0.598	0.356	1.002
Indirect Bilirubin	0.103	0.063	2.664	0.103	1.109	0.980	1.255
Total Bile Acid	−0.099	0.072	1.868	0.172	0.906	0.786	1.044
Albumin/Globulin Ratio	−0.648	0.358	3.266	0.071	0.523	0.259	1.056
Aspartate Aminotransferase	−0.016	0.013	1.476	0.224	0.984	0.958	1.010
Mitochondrial Isoenzyme of AST	−0.009	0.037	0.066	0.797	0.991	0.922	1.064
Alkaline Phosphatase	−0.002	0.008	0.036	0.850	0.998	0.983	1.014
*γ*-Glutamyl Transferase	−0.009	0.007	1.649	0.199	0.991	0.978	1.005
Glutamate Dehydrogenase	−0.069	0.058	1.419	0.234	0.933	0.833	1.046
Urea	−0.076	0.060	1.582	0.208	0.927	0.824	1.043
Retinol-Binding Protein	−0.068	0.027	6.488	0.011	0.934	0.886	0.984
Triglyceride	1.250	0.323	14.984	0.000	3.490	1.853	6.571
Length of Hospital Stay	0.028	0.037	0.555	0.456	1.028	0.956	1.106
Diabetes Mellitus (1)	1.026	0.592	3.008	0.083	2.791	0.875	8.903
Coronary Heart Disease (1)	2.073	0.769	7.261	0.007	7.945	1.760	35.872
Tumor Comorbidity (1)	0.132	0.660	0.040	0.841	1.141	0.313	4.160
Multiple Operations (1)	−0.038	0.589	0.004	0.949	0.963	0.303	3.057
Constant	12.742	7.264	3.077	0.079	3,41,733.006		

### 3.3 Construction of the prediction model

Based on the independent influencing factors screened by multivariate Logistic regression analysis, a prediction model for the decline in ADL one month after discharge in postoperative colorectal cancer patients was established. The dependent variable was whether a decline in ADL occurred one month after discharge (no = 0, yes = 1), and the independent variables were height, mean corpuscular volume, retinol-binding protein, triglyceride, and history of coronary heart disease.

The regression equation was as follows:

Logit(P)=−0.874−0.060×Height+0.102×MeanCorpuscularVolume−0.067×RetinolbindingProtein+1.065×Triglyceride+2.866×CoronaryHeartDisease

where *P* represents the probability of decline in ADL one month after discharge. The regression coefficients, standard errors, Wald *χ*^2^ values, OR values, and 95% confidence intervals of each variable in the final prediction model are shown in Table 3.

**Table 3:** Analysis of independent risk factors for self-care ability risk in colorectal cancer patients.

Variable	*B*	S.E.	Wald	Sig.	Exp(*B*)	95% CI for Exp(*B*)	
						Lower	Upper
Height	−0.060	0.024	6.150	0.013	0.942	0.898	0.987
Mean Corpuscular Volume	0.102	0.028	13.483	0.000	1.107	1.049	1.169
Retinol-Binding Protein	−0.067	0.020	10.842	0.001	0.935	0.898	0.973
Triglyceride	1.065	0.221	23.249	0.000	2.902	1.882	4.475
Coronary Heart Disease (1)	2.866	0.532	29.003	0.000	17.575	6.192	49.885
Constant	−0.874	4.179	0.044	0.834	0.417		

Multicollinearity diagnostics showed that the tolerance of each variable ranged from 0.93 to 0.99, and the variance inflation factor (VIF) ranged from 1.01 to 1.08, indicating no significant multicollinearity among the independent variables included in the model (Table 4).

**Table 4:** Test results for multicollinearity.

Item	Tolerance	Variance inflation factor (VIF)
Height	0.98	1.02
Coronary heart disease	0.94	1.06
Mean corpuscular volume	0.93	1.08
Retinol-binding protein	0.99	1.01
Triglyceride	0.97	1.03

### 3.4 Performance of the risk prediction model

**Figure 2:**
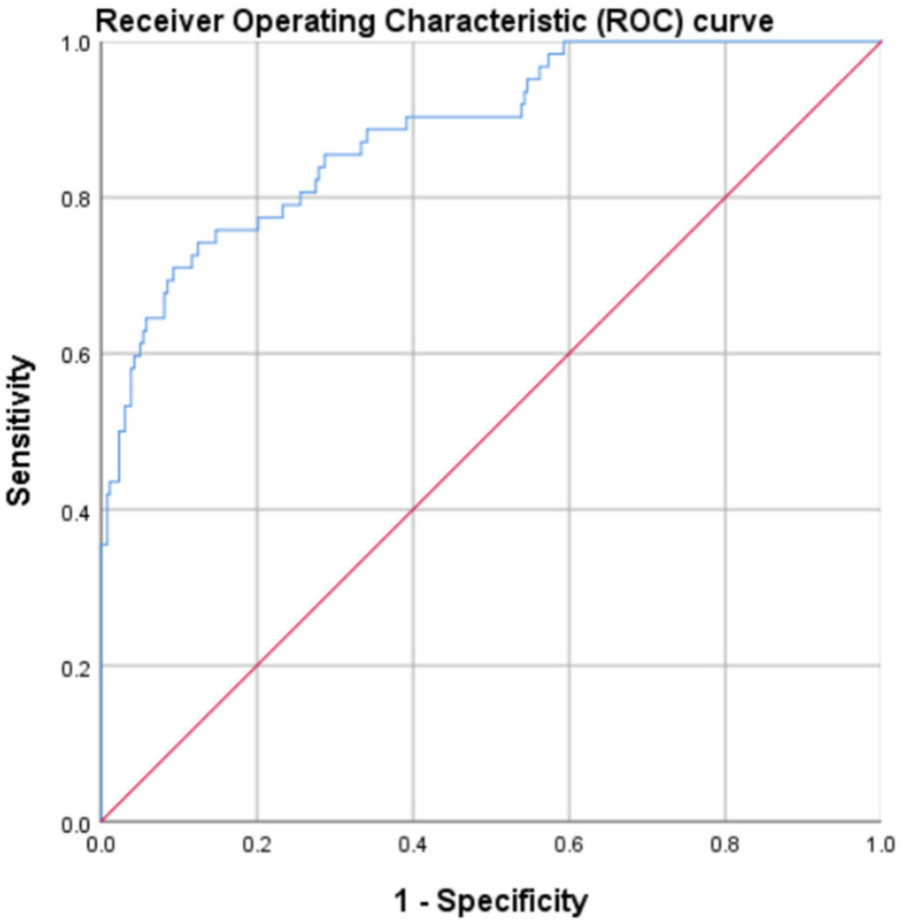
ROC curve.

The ROC curve was plotted based on the model's predicted probabilities to evaluate its predictive performance, as shown in Figure 2. The Logistic regression model achieved an AUC of 0.884 (95% confidence interval: 0.837–0.931), demonstrating excellent to superior discriminative ability with stable and reliable results. The optimal cut-off value determined by the Youden index was 0.205, with a corresponding sensitivity of 74.2%, specificity of 87.6%, precision of 59.0%, NPV of 93.4%, and F1-score of 65.8%.

**Figure 3:**
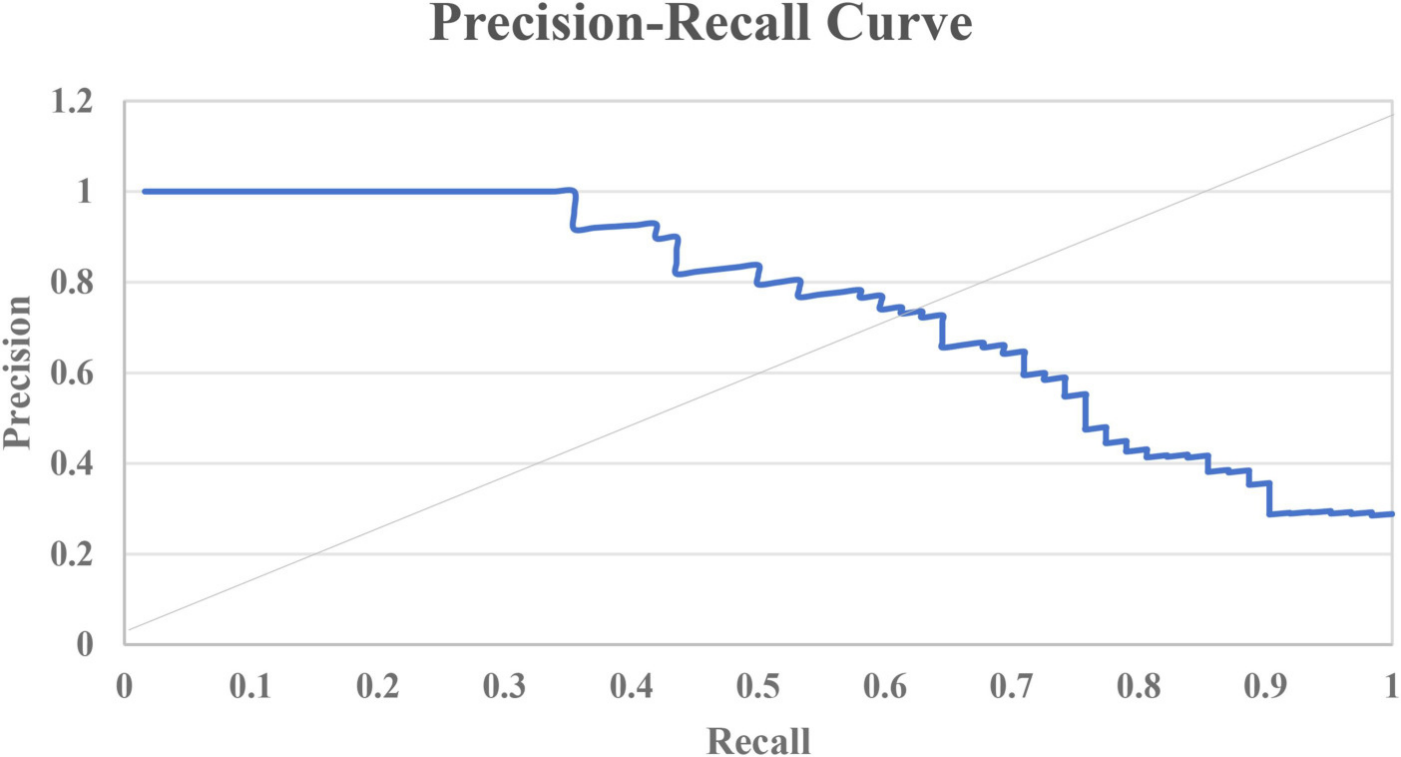
Precision-recall curve.

To address the class imbalance issue, we further constructed the PR curve (Figure 3), which yielded an Average Precision (AP) value of 0.73. This result indicates that the model maintains good predictive performance for identifying patients at high risk of ADL decline, even in the context of imbalanced data.

**Figure 4:**
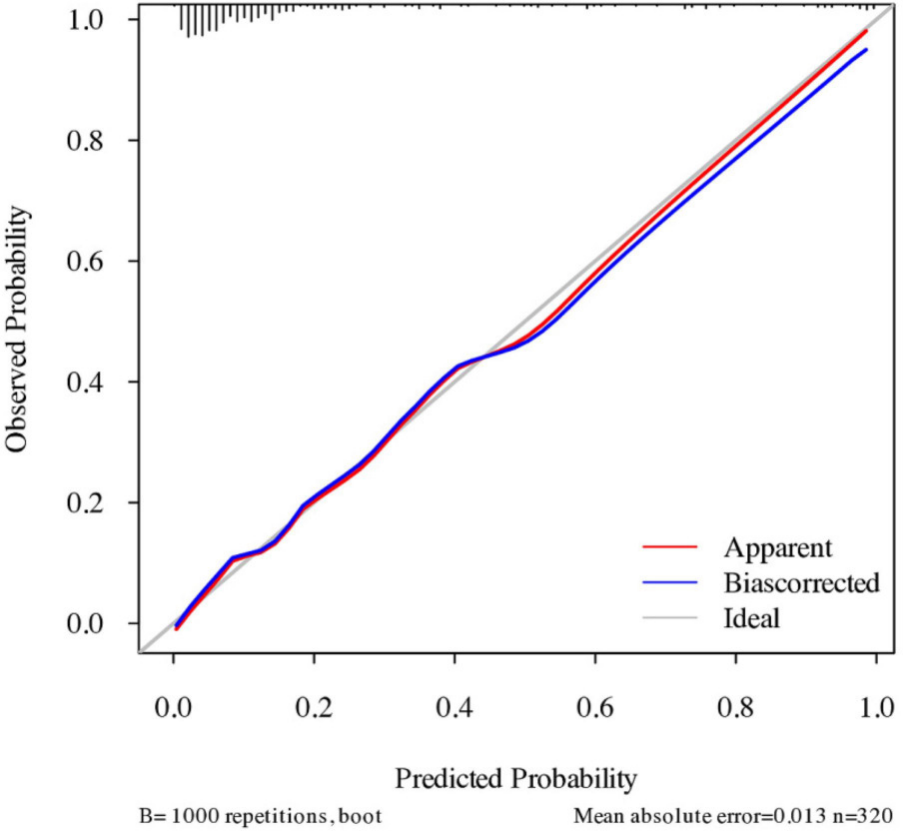
Calibration plot of the prediction model.

Model calibration was assessed using a calibration plot, calibration intercept, calibration slope, and Brier score. As shown in Figure 4 (calibration plot), the predicted probabilities agreed well with observed outcomes. The calibration intercept was 0.003, the calibration slope was 0.987, and the Brier score was 0.0884, indicating excellent calibration.

### 3.5 Internal validation and performance evaluation of the model

A total of 320 complete cases were included in this study, among whom 62 patients experienced a decline in ADL and 258 patients did not. Internal validation of the final prediction model was performed using the Bootstrap resampling method with 1,000 resampling iterations.

**Figure 5:**
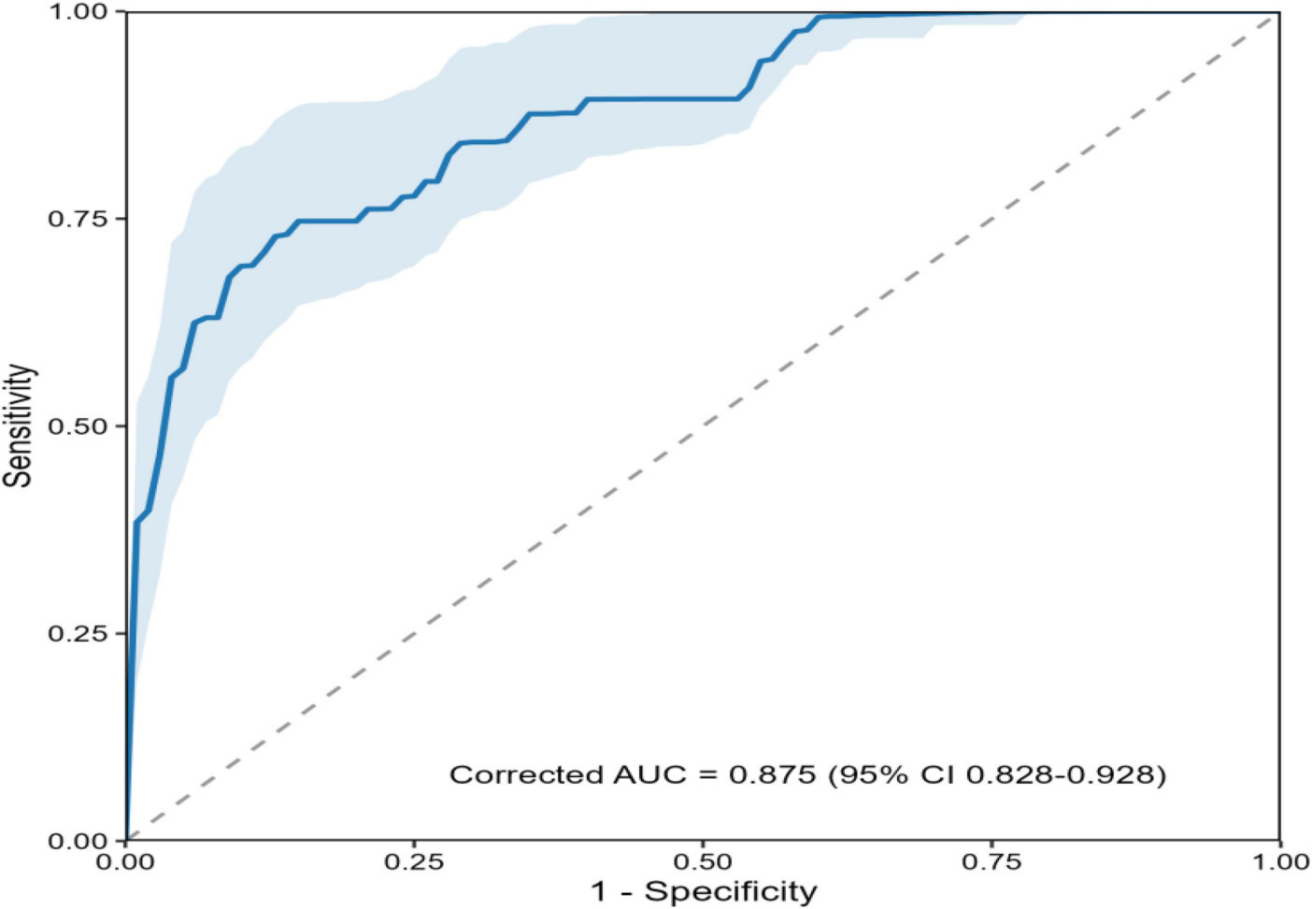
Optimism-corrected ROC curve.

The results showed that the apparent AUC of the model in the original sample was 0.884, and the AUC after optimism correction was 0.875 (95%CI: 0.828–0.928), indicating that the model still maintained good discrimination after internal correction and had a low degree of overfitting (Figure 5).

### 3.6 Sensitivity analysis

To assess whether the original model was confounded by age and sex, we forced these two variables into the multivariable logistic regression model (Enter method) together with the five original predictors. As shown in Table 5, the odds ratios (ORs) and 95% confidence intervals (CIs) of the five independent predictors (height, MCV, RBP, triglyceride, and CHD history) remained virtually unchanged compared with the original model (Table 3). Neither age (OR = 1.005, 95% CI: 0.970–1.041, *P* = 0.775) nor sex (OR = 1.436, 95% CI: 0.644–3.201, *P* = 0.376) was statistically significant. These findings indicate that the original model is robust and not substantially confounded by demographic characteristics.

**Table 5:** Test results for multicollinearity.

Predictor	Original model OR (95% CI)	Sensitivity analysis OR (95% CI)
Height	0.942 (0.898–0.987)	0.946 (0.901–0.994)
Coronary heart disease	17.575 (6.192–49.885)	19.174 (6.600–55.707)
Mean corpuscular volume	1.107 (1.049–1.169)	1.106 (1.047–1.169)
Retinol-binding protein	0.935 (0.898–0.973)	0.935 (0.897–0.974)
Triglyceride	2.902 (1.882–4.475)	2.900 (1.878–4.480)
Age	—	1.005 (0.970–1.041)
Gender	—	1.436 (0.644–3.201)

## 4 Discussion

### 4.1 Main findings and interpretation of results

This study enrolled postoperative colorectal cancer patients with a Barthel Index grade A at discharge, analyzed the factors associated with the decline in ADL at 1 month after discharge, and established a risk prediction model. The results demonstrated that although all included patients had basic independent living capacity at discharge, 62 patients still experienced varying degrees of decreased self-care ability within 1 month after discharge, with an incidence rate of 19.4%.

This finding indicates that achieving “normal self-care ability” at discharge does not mean the completion of postoperative recovery. The early post-discharge period remains a critical stage with frequent fluctuations in functional status and changes in care needs.

This finding is generally consistent with previous research trends regarding postoperative rehabilitation for malignant tumors and functional outcomes in elderly surgical patients. Aljinović et al. reported that uneventful perioperative management, controlled complications, or shortened hospital stay do not necessarily guarantee that patients will regain stable functional status after discharge. Some patients who meet clinical discharge criteria may still experience decreased mobility or increased dependence in ADL after returning home, due to insufficient physical reserve, delayed nutritional recovery, persistent chronic disease burden, and limited family care support (14).

From the perspective of postoperative recovery, the decline in ADL is not driven by a single factor, but more likely represents a comprehensive outcome resulting from the combined effects of surgical trauma, inflammatory stress, nutritional depletion, reduced exercise tolerance, and pre-existing comorbidity burden (15). During hospitalization, patients are usually under medical monitoring, symptom control, and nursing assistance, enabling them to perform basic activities in a relatively supportive environment. However, after discharge, patients must independently manage dietary adjustment, bowel care, wound healing, physical activity rehabilitation, and chronic disease control, at which point their underlying inadequate recovery and functional vulnerability become more apparent (16, 17).

In other words, achieving Barthel Grade A at discharge mainly reflects that patients have attained basic independence in the hospital setting, but does not necessarily indicate that they possess stable and sustained independent living capacity in the home environment (18).

The results of this study also suggest that postoperative functional outcomes should be regarded as an important supplementary dimension in the rehabilitation assessment of patients with colorectal cancer. Compared with traditional indicators such as complications, readmission, or length of hospital stay, ADL can more directly reflect patients' independence after returning to real life, and can better indicate whether discharge preparation is adequate, follow-up support is timely, and family care burden is increased (19). For patients who have achieved “normal” self-care ability at discharge, a subsequent short-term decline in ADL indicates that management strategies including pre-discharge risk identification, early post-discharge follow-up, and stratified intervention still need to be further optimized (20).

In addition, using short-term postoperative changes in ADL as the endpoint in this study partly addresses the current situation in perioperative colorectal cancer research, in which more attention is paid to in-hospital outcomes while relatively little attention is paid to post-discharge functional recovery (21). Postoperative rehabilitation for patients does not end with wound healing, stable laboratory parameters, or uneventful discharge. The ability to maintain basic independent living after discharge is also critical to quality of life, family burden, and subsequent rehabilitation planning. This study selected 1 month after discharge as the observation time point because this stage remains an early window of transition from postoperative recovery to home-based rehabilitation, during which patients' functional status is still largely influenced by perioperative factors and baseline reserve at discharge (22). If the observation time is extended to 3, 6, or 12 months postoperatively, functional outcomes would be more easily confounded by various out-of-hospital factors such as adjuvant therapy, disease progression, readmission, family support, and individual rehabilitation behavior, and the predictive specificity of routine pre-discharge clinical indicators would be relatively weakened (23). Therefore, using the decline in ADL at 1 month after discharge as the endpoint is more consistent with the study purpose of early risk identification based on routine pre-discharge data, and also provides a practical basis for the subsequent implementation of stratified transitional care interventions.

### 4.2 Clinical significance of independent influencing factors

Multivariate analysis showed that height, mean corpuscular volume (MCV), retinol-binding protein (RBP), triglycerides (TG), and a history of coronary heart disease (CHD) were independent influencing factors for the decline in ADL in colorectal cancer patients at 1 month after discharge. When these variables are considered collectively, they do not indicate an abnormality in a single organ, but are more consistent with a high-risk profile characterized by “insufficient recovery reserve, poor nutritional/metabolic status, and heavy burden of chronic diseases”. Previous studies have suggested that the postoperative functional outcomes of colorectal cancer patients are not only affected by the success of surgery, but also by preoperative or perioperative frailty, sarcopenia, nutritional depletion, and overall functional status, which are also closely associated with postoperative functional decline and adverse outcomes (24). In elderly patients with malignant tumors, functional decline itself has been recognized as an important and independent clinical outcome following cancer treatment (25). Meanwhile, longer duration of perioperative rehabilitation training can improve ADL performance after laparoscopic colorectal cancer surgery, which from an intervention perspective suggests a modifiable link between functional reserve and recovery capacity.

First, the inclusion of height in the final model suggests that basic physical condition may play an indirect role in short-term postoperative functional recovery. Height itself is not a modifiable factor and should not be interpreted mechanically as a direct causal variable. However, compared with body weight, height is less affected by perioperative fluid fluctuations, short-term nutritional intake, and tumor consumption, and can serve to some extent as a surrogate indicator of long-term physical development and baseline physiological reserve (26). However, it should be noted with caution that height may also reflect gender, body type characteristics, early nutritional history, or other unmeasured confounding factors.

Previous colorectal surgery studies have shown that preoperative frailty is significantly associated with postoperative functional decline, and one core component of frailty is precisely the reduction in muscle strength, endurance, and reserve capacity (24, 27). Therefore, the association between shorter height and functional decline in this study is more likely to reflect insufficient skeletal muscle reserve, exercise tolerance, and overall compensatory capacity, rather than an independent biological pathogenic effect of height itself.

Second, the independent associations of mean corpuscular volume (MCV) and retinol-binding protein (RBP) indicate that nutritional and hematological reserve status is crucial for maintaining self-care ability after surgery. Elevated MCV is commonly related to macrocytic anemia, which is often caused by nutritional factors such as vitamin B12 or folic acid deficiency, and may also indicate underlying chronic consumption, malabsorption, or altered hematopoiesis (28, 29). For patients with colorectal cancer, tumor burden, perioperative fasting, insufficient postoperative intake, and altered intestinal absorption may all exacerbate these abnormalities (30). It should be noted, however, that in this predictive model, MCV should be understood as a hematological indicator associated with adverse functional outcomes, rather than as a diagnostic criterion for confirmed nutritional deficiencies or hematopoietic abnormalities. Retrospective data cannot distinguish the specific etiology of elevated MCV and are not suitable for direct causal inferences. RBP is a nutrition-related protein with a short half-life and is sensitive to recent changes in protein-energy status (31). Yanagaki et al. reported that RBP levels in postoperative cancer patients fluctuate with changes in postoperative nutritional and metabolic status (32). Basu et al. also observed lower circulating retinol and its binding protein levels in postoperative colorectal cancer patients than in controls (33). Meanwhile, consistent evidence in colorectal surgery has demonstrated that malnutrition is associated with increased postoperative complications and poor recovery (34, 35). In the context of this study, elevated MCV and reduced RBP can be interpreted as markers of insufficient nutritional reconstitution, impaired anabolism, or weak recovery reserve. These conditions may manifest as fatigue, decreased endurance, and delayed tissue repair, ultimately leading to short-term decline in ADL after discharge. However, it should be noted with caution that a decrease in RBP does not equate to confirmed malnutrition; in predictive models, it should be understood more as an auxiliary indicator related to functional recovery rather than an independent pathogenic factor.

The inclusion of triglycerides in the final model indicates that metabolic abnormalities may also be involved in postoperative functional recovery. Zhang et al. demonstrated that metabolic syndrome is associated with ADL dependence, functional limitation, and physical decline (36). Recent longitudinal studies also suggest that triglyceride-related metabolic indices such as the TyG index are linked to ADL disability (37). This implies that elevated triglycerides may not simply reflect an abnormal lipid level, but rather a broader state of metabolic imbalance, including insulin resistance, chronic low-grade inflammation, visceral fat accumulation, and reduced exercise tolerance (38). For colorectal cancer patients who have just undergone surgical trauma, such metabolic disorders may further impair physical rehabilitation, activity recovery, and chronic disease control, thereby increasing the risk of post-discharge decline in ADL.

The inclusion of a history of coronary heart disease in the final model indicates that patients with coronary heart disease are more susceptible to limitations in independent living and functional performance. Frailty, sarcopenia, and multimorbidity are highly prevalent in elderly patients with coronary artery disease (CAD), making them more vulnerable in terms of functional recovery and treatment tolerance (39). Restricted coronary blood supply reduces exercise tolerance and increases fatigue and discomfort after physical activity, while long-term cardiovascular burden is often associated with deconditioning, activity avoidance, and global functional decline (40, 41). For patients after colorectal cancer surgery, this means that even if they achieve Barthel Grade A at discharge, they may still develop rapid functional decline in activities with real daily physical demands—such as climbing stairs, walking, and toilet transfer—due to insufficient cardiovascular reserve after returning home. This result also suggests that discharge assessment should not focus only on the surgical site and the tumor itself, but should also integrate chronic disease burden, particularly cardiovascular comorbidities, into the framework of functional risk identification.

From a clinical perspective, these independent influencing factors do not exist in isolation but collectively indicate early postoperative functional vulnerability. This also suggests that the maintenance of self-care ability after discharge in patients with colorectal cancer depends not only on surgical recovery itself but also on the continuous effects of baseline reserve, nutritional and metabolic status, and chronic disease burden. However, it must be reiterated that the aforementioned interpretation is based on statistical correlations derived from retrospective predictive models and should not be regarded as direct evidence of causal mechanisms.

### 4.3 Performance and value of the prediction model

This study established a prediction model for the decline in ADL at 1 month after discharge in postoperative colorectal cancer patients based on height, mean corpuscular volume (MCV), retinol-binding protein (RBP), triglycerides, and a history of coronary heart disease. The results showed that the apparent AUC of the model in the original dataset was 0.884, and the corrected AUC after Bootstrap internal validation was 0.875, indicating favorable discrimination, minimal optimism bias, and satisfactory internal stability. Combined with the calibration curve results, the predicted probabilities of the model were generally consistent with the actual observed probabilities, suggesting that the model achieved good fitting performance and predictive accuracy in the study population.

Compared with previous perioperative studies in colorectal cancer that mostly focused on predictive outcomes such as postoperative complications, readmission, length of hospital stay, or long-term survival, the model constructed in the present study targeted early post-discharge decline in ADL, a functional outcome that more closely reflects actual rehabilitation status (42). Although such an outcome is not directly equivalent to traditional medical endpoints, it better reflects patients' independent living ability and recovery quality after returning to the home environment. In this sense, this study complements previous perioperative risk assessment studies in terms of predictive outcomes, and also enables the model to be more directly applied to discharge preparation and transitional care management.

In terms of model composition, all predictors included were derived from routinely available clinical data before discharge. Their combination, to some extent, comprehensively reflects patients' baseline physical reserve, nutritional and metabolic status, as well as chronic disease burden. The integration of such multidimensional information may represent an important reason for the model's favorable discriminative performance. In other words, the predictive power of the model does not rely mainly on a single strong indicator, but is based on the joint representation of the underlying state of “early postoperative functional vulnerability” by multiple routine variables. This also suggests that short-term post-discharge decline in ADL cannot be explained by a single complication or isolated laboratory abnormality, but is more likely a concentrated manifestation of insufficient perioperative recovery reserve in the post-discharge setting.

From a clinical application perspective, the advantage of this model is that all required variables can be routinely obtained before discharge, without additional complex examinations, specialized equipment, or dedicated scale assessments, thus yielding favorable accessibility and practical feasibility. For postoperative colorectal cancer patients, identifying those at high risk of functional decline within 1 month after discharge prior to hospital discharge allows more targeted and proactive allocation of limited medical and nursing resources to patients who truly need intensive management. Specifically, for patients identified as high-risk by the model, clinical strategies can be strengthened, including enhanced discharge education, nutritional support recommendations, physical and rehabilitation guidance, coordinated chronic disease management, and caregiver preparation, thereby improving the targeting of early post-discharge follow-up and interventions.

Furthermore, the value of this model lies not in replacing clinical judgment, but in providing a relatively objective and operable risk stratification tool for healthcare professionals. In routine clinical practice, discharge eligibility is often determined mainly based on stable disease status, wound healing, and recovery of basic functional ability during hospitalization. However, simple and quantitative evidence is lacking for evaluating patients' potential to maintain independent living after returning home. The model in this study was developed to address this gap, making it more suitable as a supplementary tool for discharge assessment rather than a sole decision-making criterion. For nursing practice, such a prediction tool based on routine data can also facilitate the transition of transitional care management from universal follow-up to stratified follow-up, enabling high-risk patients to receive closer monitoring and more targeted support in the early postoperative period.

## 5 Limitations of the study

This study has several limitations. First, as a single-center retrospective study, the sample source and perioperative management protocol were institution-specific. Although internal validation was performed using the Bootstrap method, the external generalizability of the model remains to be further verified in more regions and independent populations. Second, candidate variables were mainly derived from routine clinical data obtained before discharge. Some important factors potentially affecting postoperative functional recovery, such as frailty, sarcopenia, severity of postoperative complications, family care capacity, and rehabilitation adherence, were not included in the analysis, which may result in residual confounding. This study did not incorporate key variables that may influence postoperative functional recovery, such as perioperative severe complications, postoperative adjuvant chemotherapy, surgical approach, stoma creation, discharge destination, frailty status, sarcopenia, family caregiving capacity, and rehabilitation training adherence. Due to limitations in data availability, these factors were not included in the multivariate adjustment, which may introduce residual confounding. Future studies could further incorporate these variables to refine model construction and risk interpretation.

In addition, this study defined the outcome as a decline in the Barthel Index from Grade A to Grades B–F at 1 month after discharge, focusing on early postoperative functional decline. While this design facilitates early risk identification, it did not further distinguish between different levels of dependence nor cover longer-term functional changes. The model established in this study is mainly intended for early risk screening before discharge. Future multi-center prospective studies are warranted for external validation, to optimize the model by incorporating more functional recovery–related indicators, and to evaluate the real-world effect of targeted interventions based on this risk stratification. Furthermore, due to limitations in sample size and the exploratory nature of this study, no further development of a simplified risk scoring tool or risk stratification chart was conducted, nor was decision curve analysis performed; these aspects will be supplemented in subsequent multicenter external validation studies. We also acknowledge that the linearity assumption for continuous predictors was not formally tested, which is a limitation of our analysis.

## Data Availability

The raw data supporting the conclusions of this article will be made available by the authors, without undue reservation.
